# “Mortgage renegotiation, monetary gains and financial literacy: A dataset of Chilean households”

**DOI:** 10.1016/j.dib.2021.107553

**Published:** 2021-11-07

**Authors:** Carlos Madeira

**Affiliations:** Central Bank of Chile, Chile

**Keywords:** Household finance, Mortgage refinancing, Financial portability, Financial literacy

## Abstract

This article provides data on the Mortgage Renegotiation behavior of a sample of Chilean households, plus estimates of the Present Value of the gains of mortgage refinancing and the number of past months in which it was optimal to make a refinancing choice. Furthermore, I impute the value of the Financial Literacy index of each household, according to the OCDE-INFE methodology. This data is useful for academics, policy makers and business analysts interested in the relationship between mortgage refinancing and financial literacy.

## Specifications Table

**Table:** 

Subject	Economics, Econometrics and Finance.
Specific subject area	Household Finance. Financial Literacy.
Type of data	Table (Excel format and Stata dta files)
How data were acquired	Data combines publicly available raw data from the Chilean Household Finance Survey (EPF, 2007-2017 waves) and the Chilean Financial Capabilities Survey (ECF, 2016 wave). Hardware: data analysis was performed in a standard notebook with an Intel Core i7-4700HQ 2.40 GHz processor with 16.0 GB of RAM. Software: Stata MP-6 (version 15.1).
Data format	Analyzed (EFH_mortgage_renegotiation.xlsx – same file provided in Stata format).
Parameters for data collection	The main data are obtained from the sample of the Chilean Household Finance Survey (EFH), with information on demographics and refinancing behavior. For each household in the sample, I then impute the Financial Education indexes based on a linear regression using the respondents’ gender, age (10 year dummies), education (5 levels of education), and dummies for the quartile of the household income. The Financial Literacy indexes correspond to the OECD-INFE methodological standards.
Description of data collection	Data consists of 2 overall Financial Literacy indexes, with 4 sub-indexes.
Data source location	Central Bank of Chile, Santiago, Chile. Financial Market Commission, Santiago, Chile. https://www.efhweb.cl/ (EFH) https://scioteca.caf.com/handle/123456789/985 (ECF)
Data accessibility	With the article.
Related research article	Madeira, C. (2021), “The potential impact of financial portability measures on mortgage refinancing: Evidence from Chile,” *Journal of International Money and Finance*, 117, 102455.

## Value of the Data

• The data can be used to study how mortgage refinancing in Chile is related to Financial Education, demographics and the pecuniary gains associated with the refinancing option.
• The data includes 6088 households observed in different years (2007, 2008, 2009, 2010, 2011, 2014, 2017) and with different sex-age-education-income demographics, therefore allowing for an analysis of how mortgage refinancing evolved in Chile over the years across heterogeneous groups of households.
• For each household in the sample, the data shows both two overall Financial Literacy indexes and 4 sub-indexes (corresponding to Search, Financial Knowledge, Financial Behavior, and Financial Attitudes). These variables are formatted according to the OCDE-INFE methodology and can therefore be compared with other countries. The possibility for international comparison allows researchers to evaluate how Chile compares to European countries and the United States in terms of financial education and mortgage refinancing behavior.
• The dataset includes the Present Value of the Mortgage Refinancing Gains and the Number of Months for Refinancing. This allows to calculate the welfare gains for the population of borrowers in Chile for changes made to the mortgage credit laws.

## 1 Data Description

One data files are included: EFH_mortgage_renegotiation.xlsx. The same dataset is available in Stata: EFH_mortgage_renegotiation.dta. The dataset includes 21 variables. The variables folio_efh (Household ID) and year_efh (Survey Wave Year) can be used to match with other information that interested researchers can obtain from the Chilean Household Finance Survey (EFH) dataset. This dataset provides information on: i) the demographics of the households (sex, age, education, income); ii) their financial education sub-indexes (Financial Attitudes, Financial Behavior, Financial Knowledge, and Search Behavior for Financial Information) and overall financial literacy indexes (the sum of all the 4 sub-indexes; the sum of Behavior, Knowledge and Search sub-indexes); iii) Mortgage Refinancing Behavior (whether the household has refinanced a mortgage in the past, the Present Value of Refinancing, the Number of Past Months with a positive Refinancing opportunity).

## 2 Experimental Design, Materials and Methods

To build the dataset I use the Chilean Household Finance Survey (in Spanish, *Encuesta Financiera de Hogares*, hence on EFH), which is a representative national survey with detailed information on assets, debts, income and financial behavior. The EFH is comparable to similar surveys in the United States and Europe, such as the Survey of Consumer Finances (SCF) and the Household Finance and Consumption Survey (HFCS). The seven EFH survey waves (2007, 2008, 2009, 2010, 2011, 2014, 2017) covered 21,319 urban household interviews, with an over representation of richer households (which is common also in other countries). This dataset includes only the 6,088 households which report having at least one mortgage being repaid at the time of the interview.

The EFH survey has little information on financial education. However, the Survey of Financial Capabilities (in Spanish, *Encuesta de Capacidades Financieras*, hence on, ECF) measured in 2016 an extensive set of financial literacy indexes for 1224 Chilean households, using the same methodology applied to other members of the OECD / INFE (International Network on Financial Education) network (Atkinson and Messy [3]). It is therefore possible to impute the financial literacy indexes for each EFH respondent using the mean indexes of similar ECF individuals, based on age (10 year dummies), gender, education level and household income quintile. Table 1 shows the mean levels of 4 different financial literacy indexes: Financial Attitudes, Financial Behavior, Financial Knowledge, and Search Behavior for Financial Information. Financial Attitudes measures on a scale from 0 to 5 whether households prefer to spend money instead of saving it. Financial Behavior measures on a scale from 0 to 8 a set of behaviors such as thinking before making a purchase, paying bills on time, budgeting, saving or borrowing to make ends meet. Financial Knowledge measures on a scale from 0 to 8 the basic knowledge of a range of financial topics, such as division, risk-return trade off, inflation, interest rates, and asset diversification. The Search Behavior for Financial Information measures on a scale from 0 to 3 measures how much information the household gathers on different financial products and financial institutions, the diversity of its information sources on financial products (internet, financial advisors whether in person or by phone, friends, newspapers...) and how frequently the household has borrowed over the last year.

**Table 1:** Description of the analyzed datasets (EFH_mortgage_renegotiation.xlsx, plus its Stata .dta version) provided in this article.

Analyzed dataset	Description
*EFH_mortgage_renegotiation.xlsx (EFH_mortgage_renegotiation.dta)*	*Information on a sample of 6,088 Chilean households, with detail on their demographics, Financial Education Indexes, and Mortgage Refinancing Behavior.*

In the original definition of Financial Literacy of the OECD / INFE network, Education was the sum of all the Attitude, Behavior, Knowledge and Search indexes (Atkinson and Messy [3], OECD [4]). In developed economies, the Attitude questions are related to households that prefer ``savings'' over ``debt'' financial products (OECD [4]). Since in Chile the Attitude index is more related to the conservative spending background of the low income and less educated households, then I provide a second overall Financial Education Index that is just the sum of the Behavior, Knowledge and Search sub-indexes.

Furthermore, I computed both the Present Value of the Mortgage Refinancing for each household and the Number of Months in which a positive Refinancing opportunity was available to the household. These variables are calculated using the optimal closed-form solution in Agarwal et al. [2], which applies a real-option model to derive a closed formula for the interest rate value at which a household should refinance its mortgage and also for the present value of the gains of refinancing. This formula considers that refinancing a mortgage is a real-option that a rational borrower unconstrained by credit frictions can exercise before the end of the credit. The model accounts in a non-linear way for the Current interest rate, the Contractual interest rate (fixed) of the mortgage, the Maturity (in years) of a new mortgage contract after refinancing, the (nominal) debt of the mortgage contract, the reremaining maturity (in years) of the mortgage contract, the marginal tax rate faced by the households, the inflation rate, the real discount rate, the annual standard-deviation of the mortgage real interest rate, the exogenous annual probability of a household moving to another home, the exogenous annual probability of refinancing, the variable costs and the fixed costs of refinancing proportional to the loan value.

All the methods (in Stata do-files) are published in the RunMyCode companion website of the original research article (Madeira [1]).

## CRediT Author Statement

**Carlos Madeira:** Conceptualization, Methodology, Software, Validation, Formal analysis, Investigation, Resources, Data curation, Writing – original draft, Writing – review & editing, Visualization, Supervision, Project administration, Funding acquisition.

## Declaration of Competing Interest

The author declares that he has no known competing financial interests or personal relationships which have or could be perceived to have influenced the work reported in this article. I received no funding from any institution besides my employer which is the Central Bank of Chile. Furthermore, there are no patents or impediments to publication, including the timing of publication, with respect to the intellectual property of the article or the associated dataset.

## Appendix Supplementary materials

Image, application 1

Image, application 2

## References

[1] Madeira C. (2021). The potential impact of financial portability measures on mortgage refinancing: evidence from Chile. J. Int Money Finance.

[2] Agarwal S., Driscoll J., Laibson D. (2013). Optimal mortgage refinancing: a closed-form solution. J. Money Credit Bank..

[3] Atkinson A., Messy F. (2012).

[4] (2020).

